# Fostering a culture of inquiry through a dedicated Nursing Research Clinic: a mixed-methods evaluation of the CREATE model

**DOI:** 10.3389/fmed.2026.1738330

**Published:** 2026-06-09

**Authors:** Yinan Liu, Yinyin Lü, Ning Ma, Caihong Li, Yuan Ke, Hongming Guo

**Affiliations:** 1 Department of Palliative Care, Beijing Tsinghua Changgeng Hospital, School of Clinical Medicine, Tsinghua University, Beijing, China; 2 Department of Nursing, Beijing Tsinghua Changgeng Hospital, School of Clinical Medicine, Tsinghua University, Beijing, China; 3 Department of Case Management, Beijing Tsinghua Changgeng Hospital, School of Clinical Medicine, Tsinghua University, Beijing, China; 4 Department of Operating Room, Beijing Tsinghua Changgeng Hospital, School of Clinical Medicine, Tsinghua University, Beijing, China

**Keywords:** academic-practice partnerships, clinical inquiry, evidence-based practice, mentorship models, nursing research capacity, organizational culture, research competence

## Abstract

**Background:**

Despite widespread recognition of evidence-based practice as fundamental to quality healthcare, persistent theory-to-practice gaps undermine optimal patient outcomes. Structural barriers including inadequate mentorship, limited resources, and organizational constraints prevent clinical nurses from engaging meaningfully in research activities.

**Objective:**

To evaluate the observed changes in clinical nurses’ research competencies and inquiry cultures associated with a novel Nursing Research Clinic operationalized through the CREATE (Consultation, Review, Enlighten, Advice, Ticket, Enhancement) model.

**Design:**

Convergent parallel mixed-methods design employing a single-group pre-post intervention assessment with qualitative exploration of participant experiences.

**Methods:**

Following institutional ethics approval, 86 registered nurses with ≥1-year clinical experience participated in the CREATE intervention at Beijing Tsinghua Changgeng Hospital (October 2023–October 2024). Perceived research competence was assessed pre- and post-intervention using the Nursing Research Competence Self-Assessment Scale (NRCSAS), a 30-item instrument adapted for clinical nurses from a previously published research competency scale. Research productivity metrics were tracked, and semi-structured interviews with 20 participants explored experiences through inductive thematic analysis.

**Results:**

Participants demonstrated significant improvements across all competence domains, with total scores increasing from 68.72 ± 18.38 to 84.97 ± 12.95 (*p* < 0.001). Effect sizes ranged from medium to large (Cohen’s *d* = 0.45–1.47), with largest improvements in data processing (*d* = 1.47) and research practice (*d* = 1.09). Early-stage productivity outcomes included one core journal publication, three national presentations, three patents, and four funded projects. Qualitative analysis revealed five themes: transformation from apprehension to empowerment, catalytic mentorship role, legitimization through institutional recognition, enhanced collaboration, and navigation of research challenges.

**Conclusion:**

The CREATE model represents a structured and replicable intervention that systematically addresses barriers to nursing research engagement. This evaluation demonstrates that the model is associated with meaningful improvements in research competence and early-stage scholarly productivity, supported by qualitative evidence of professional identity transformation, mentorship effectiveness, and institutional legitimization. Future multi-site-controlled studies are recommended to further establish generalizability and long-term sustainability.

## Introduction

The integration of evidence-based practice (EBP) represents a fundamental cornerstone of contemporary healthcare delivery, synthesizing the best available research evidence with clinical expertise and patient preferences to optimize care outcomes and enhance professional accountability (1). Within nursing practice specifically, systematic EBP adoption has demonstrated meaningful benefits including enhanced patient safety profiles, improved care process efficiency, reduced healthcare expenditures, and strengthened professional autonomy and decision-making capabilities (2). International accreditation bodies, most notably the Magnet Recognition Program^®^ administered by the American Nurses Credentialing Center, have consequently established robust EBP implementation as a mandatory criterion for organizational nursing excellence, reflecting the global professional consensus regarding its indispensable role in contemporary quality healthcare delivery (3).

Nevertheless, a substantial and persistent chasm continues to exist between nursing research generation and its consistent clinical application–the widely recognized and extensively documented “theory-to-practice gap” or “research-practice divide (4).” This fundamental disconnect means that clinical decisions frequently rely upon tradition, institutional precedent, and practitioner intuition rather than rigorous scientific evidence, potentially compromising care quality and significantly impeding nursing’s continued evolution and maturation as a fully integrated scientific discipline (5). A recent mixed-methods systematic review by Morrison et al. synthesized evidence on factors influencing research activity among nurses in clinical practice, identifying persistent barriers including lack of time, confidence, mentorship access, and organizational support across diverse healthcare settings (6). These findings reinforce the urgency of developing structural interventions that simultaneously address individual and organizational barriers to research engagement. In China, where nursing achieved formal recognition as a first-level academic discipline within the national educational framework in 2026 (7), this challenge has assumed particular significance and urgency. Despite substantial governmental and institutional investments in nursing education infrastructure, faculty development, and research capacity building initiatives, the field continues to contend with insufficient research capability among clinical personnel, suboptimal research methodological quality, and disappointingly low rates of successful research translation into sustainable practice innovations (8).

The persistent failure to consistently integrate evidence into routine clinical practice stems not from inadequate motivation or intellectual deficiencies among frontline nursing practitioners, but rather from a complex constellation of deeply entrenched and mutually reinforcing systemic barriers operating at multiple organizational levels (9). Organizational obstacles predominate within this barrier complex, including overwhelming clinical workload demands that preclude meaningful time allocation for scholarly activities, inadequate infrastructural resources such as research funding, database access privileges, statistical consultation services, and institutional absence of protected research time or sabbatical opportunities (10). These structural deficiencies directly contribute to individual-level barriers, where practicing nurses consistently report significant knowledge deficits in fundamental research methodologies, statistical analysis techniques, manuscript preparation protocols, and critical appraisal competencies (11). This knowledge deficit systematically fosters diminished professional confidence and pronounced reluctance to engage proactively in EBP initiatives, creating a self-perpetuating cycle of research avoidance and professional stagnation.

While numerous educational interventions have been implemented across diverse healthcare settings–ranging from traditional didactic workshop approaches to innovative experiential learning models incorporating simulation and problem-based methodologies–a critical “articulation gap” frequently emerges when nurses attempt independent research practice following formal training program completion (12). This phenomenon reveals a fundamental systems-level insight: individual knowledge deficits frequently represent consequences rather than primary causes of systemic organizational failures to invest comprehensively and sustainably in nursing professional development infrastructures. Educational interventions focused exclusively on short-term knowledge transfer, without simultaneously addressing underlying structural, cultural, and resource-related contexts, are consequently destined for limited effectiveness and poor long-term sustainability (13). This understanding aligns with Senge’s organizational learning theory, which posits that sustainable change within complex organizations requires the cultivation of shared vision, systems thinking, team learning, and personal mastery–principles that informed the design of the intervention described herein (14).

Recognizing this multifaceted challenge, and responding proactively to the expressed daily consultation needs articulated by clinical researchers throughout the institution, the Department of Nursing at Beijing Tsinghua Changgeng Hospital developed and implemented a structural intervention: the Nursing Research Clinic. This initiative moves beyond traditional training paradigms by establishing a permanent, institutionally embedded infrastructure specifically dedicated to fostering nursing inquiry, scholarship development, and sustainable research culture transformation. The clinic’s comprehensive operations are guided by a novel conceptual framework–the CREATE (Consultation, Review, Enlighten, Advice, Ticket, Enhancement) model, developed for the present study to systematically address both individual competency gaps and organizational barriers to research engagement through structured mentorship programming, comprehensive resource provision, and sustained longitudinal support mechanisms. This investigation presents a comprehensive mixed-methods evaluation of the observed changes associated with the CREATE model in enhancing research capabilities and cultivating sustainable cultures of scholarly inquiry among clinical nursing practitioners within a major academic medical center environment.

## Materials and methods

### Study design and theoretical foundation

The study was approved by the Beijing Tsinghua Changgeng Hospital Institutional Review Board and conducted in accordance with the Declaration of Helsinki. Written informed consent was obtained from all participants prior to enrolment. This investigation employed a convergent parallel mixed-methods design, incorporating simultaneous quantitative and qualitative data collection and analysis procedures conducted within identical temporal parameters (15). The quantitative research strand utilized a single-group pre-post intervention design to evaluate observed changes in participants’ self-reported research competency profiles, with the recognition that such instruments capture perceived rather than objectively demonstrated ability, while the qualitative strand employed phenomenological inquiry approaches to explore participants’ lived experiences and provide rich contextual understanding of the intervention’s underlying mechanisms of action. The study design was grounded theoretically in organizational learning theory frameworks and academic-practice partnership models, which collectively informed both intervention architecture and comprehensive evaluation approaches (14).

### Setting and organizational context

This investigation was conducted at Beijing Tsinghua Changgeng Hospital, tertiary academic medical center affiliated with Tsinghua University School of Clinical Medicine in Beijing, China. The institution functions as a major teaching facility serving approximately 2,800 nursing personnel distributed across 45 specialized clinical departments, maintaining nationally recognized programs in clinical excellence, healthcare innovation, and translational research. The Department of Nursing had previously established formal organizational commitments to evidence-based practice implementation and systematic research capacity development, thereby providing a supportive institutional context conducive to successful intervention implementation and sustainable culture transformation.

### The CREATE model intervention framework

The CREATE model represents a comprehensive practice-oriented operational framework systematically operationalizing research capacity building through six interconnected phases: Consultation (structured entry mechanism via electronic platform scheduling), Review (expert analysis and feasibility evaluation), Enlighten (creative thinking expansion and perspective broadening), Advice (intensive mentorship and protocol development), Ticket (longitudinal follow-up appointment scheduling), and Enhancement (continuous evaluation and systematic improvement processes).

The intervention was delivered by a carefully selected and extensively trained multidisciplinary team comprising 13 specialist members: one clinical director (Ph.D.-prepared nurse researcher with extensive methodological expertise), five visiting specialists (including research methodologists, biostatisticians, and clinical content experts), six master’s-level graduate research coordinators, and one dedicated administrative coordinator. All team members completed standardized training protocols in evidence-based mentorship principles, structured consultation techniques, and comprehensive CREATE framework implementation prior to intervention delivery.

The clinic operated through a purpose-built electronic platform enabling nurses to schedule appointments conveniently, submit detailed research questions, and access comprehensive educational resources. Individual consultation sessions were conducted through both face-to-face and virtual modalities, with session duration ranging from 45 to 90 min depending upon project complexity and individual learning needs. Longitudinal follow-up support was provided through systematically scheduled appointments, comprehensive email correspondence, and unrestricted access to institutional research resources including specialized databases, statistical software packages, and professional manuscript preparation assistance.

### Participants and recruitment procedures

Inclusion criteria:

- Licensed registered nurses employed full-time at the study institution
- Minimum 1 year of documented clinical experience in direct patient care
- Active engagement in direct patient care responsibilities
- Voluntary informed consent to participate in all research activities
- Commitment to complete both pre- and post-intervention assessment protocols

Exclusion criteria:

- Nurses employed primarily in administrative roles without direct patient care responsibilities
- Temporary or contract employees with less than 6 months remaining employment commitment
- Previous participation in formal research training programs within 12 months prior to enrollment
- Current enrollment in graduate degree programs with substantial research methodology components

Sample size determination: an *a priori* power analysis was conducted using G*Power 3.1, drawing on effect sizes reported in prior educational interventions targeting nursing research competence (16). A minimum sample size of 80 participants was required to detect a moderate effect size (Cohen’s *d* = 0.5) with 80% statistical power and a two-tailed Type I error rate of α = 0.05. Accounting for potential 10% participant attrition, target recruitment was established at 88 participants. No internal pilot study was conducted at the host institution prior to the main investigation; the parameters used in the power analysis were derived solely from published comparator studies. The pilot testing referenced elsewhere in this manuscript pertains exclusively to refinement of the qualitative interview guide and is not equivalent to a quantitative pilot of the intervention itself.

Recruitment strategy: participant recruitment occurred through multiple complementary strategies including comprehensive departmental presentations, targeted electronic announcements, and systematic peer referral networks. Interested nursing personnel attended detailed information sessions where study procedures, time commitment requirements, potential professional benefits, and confidentiality protections were thoroughly explained. Written informed consent emphasizing voluntary participation and unrestricted right to withdraw without penalty or professional consequence was obtained from all enrolled participants.

### Data collection procedures and instrumentation

#### Quantitative data collection

Primary outcome assessment: perceived research competence was assessed using an adapted 30-item self-assessment instrument based on the Research Competency Scale for Nursing Students developed by Qiu et al. The adapted version was contextualized for registered clinical nurses by an expert panel comprising three doctorally prepared nurse researchers and is referred to in this manuscript as the Nursing Research Competence Self-Assessment Scale (NRCSAS). The instrument retains the original six-domain architecture, measuring problem identification and formulation (5 items), literature access and critical appraisal (8 items), research design and methodology (6 items), research practice implementation (7 items), data processing and statistical analysis (5 items), and scientific writing and dissemination (4 items). The original Qiu et al. scale demonstrated robust psychometric properties in its validation study (Cronbach’s α = 0.94 for the total scale and α = 0.76–0.89 for individual subscales, Content Validity Index = 0.92, and test-retest reliability *r* = 0.88 over 2-weeks intervals); no separate revalidation was undertaken for the adapted version used in the present investigation, which represents a study limitation discussed below. Individual items are rated using a 5-point Likert scale (1 = completely incompetent to 5 = completely competent), with higher composite scores indicating greater perceived research competence (17).

Administration protocol: the NRCSAS was administered electronically using the secure REDCap database system at baseline (within 1 week prior to initial clinic consultation) and immediately following intervention phase completion (defined as final scheduled consultation or 6 months post-initial consultation, whichever occurred first). Electronic administration ensured optimal data integrity, participant anonymity protection, and systematic response completeness monitoring.

Secondary outcome measures: comprehensive research productivity metrics included systematic tracking of peer-reviewed publications, professional conference presentations, patent applications, competitive grant submissions and funding award, and quality improvement project implementations. Data were collected from institutional databases, verified participant self-reports, and external database confirmation. Additional clinic satisfaction assessment utilized a 15-item questionnaire specifically developed for this investigation, evaluating participant perceptions of service quality, mentor effectiveness, resource adequacy, and overall programmatic experience using 5-point rating scales.

#### Qualitative data collection

Participant selection strategy: purposive sampling methodology was employed to achieve maximum demographic variation in participant characteristics, clinical specialty representations, research experience levels, and intervention engagement patterns. Recruitment continued systematically until theoretical saturation was achieved, as determined when three consecutive interviews yielded no new codes or thematic variations, documented through ongoing saturation monitoring during data collection.

Interview protocol development: semi-structured interview protocols lasting 45–75 min were conducted by extensively trained research personnel using standardized interview guides. The comprehensive guide was developed through systematic literature review, expert panel consultation, and pilot testing with three nurses not participating in the primary investigation. Interview domains encompassed: detailed experiences with CREATE model processes, perceived professional benefits and encountered challenges, mentorship relationship dynamics, organizational support perceptions, persistent barriers identification, and systematic improvement recommendations.

Interview implementation: all interviews were conducted in private, comfortable settings at participant convenience, utilizing either secure in-person or encrypted video conferencing modalities. Complete audio recording was performed with explicit participant permission, followed by professional verbatim transcription and comprehensive accuracy verification. All transcripts were systematically de-identified prior to analytical procedures.

### Statistical analysis

#### Quantitative analysis

Comprehensive data management utilized the secure REDCap (Research Electronic Data Capture) system hosted at the study institution. Descriptive statistics were calculated for all variables, including frequency distributions and percentages for categorical variables, and means with standard deviations for continuous measures. Distribution normality was assessed using Shapiro-Wilk statistical tests and visual histogram inspection.

Pre-post competence score comparisons were analyzed using paired *t*-tests for normally distributed data and Wilcoxon signed-rank tests for non-normally distributed variables. Effect sizes were calculated using Cohen’s d with 95% confidence intervals, with statistical significance established at *p* < 0.05 for all two-tailed tests. Multiple comparison corrections were applied using Bonferroni methodology for subscale analyses. Missing data were managed using multiple imputation methods when less than 10% of data points were absent, with sensitivity analyses conducted excluding participants with missing data to assess finding robustness.

Exploratory subgroup analyses were conducted to examine whether improvement patterns differed according to prior research experience. Independent samples *t*-tests were employed to compare mean change scores between participants with and without prior research experience, with corresponding effect sizes calculated using Cohen’s d.

#### Qualitative analysis

Qualitative data were analyzed using Braun and Clarke’s comprehensive six-phase thematic analysis framework (18). An inductive coding approach was adopted, whereby codes were derived from the data themselves rather than from a pre-existing theoretical framework, ensuring that the analysis remained grounded in participants’ lived experiences. The analytical team consisted of three experienced researchers with demonstrated expertise in qualitative methodologies, nursing research, and organizational behavior analysis. Initial coding procedures were conducted independently by two researchers, with discrepancy resolution achieved through systematic discussion and consensus development.

Analytical process: Phase 1 involved comprehensive data familiarization through repeated transcript reading and annotation. Phase 2 included systematic coding of meaningful analytical units using NVivo software version 12. Phase 3 focused on thematic pattern identification across generated codes, with initial codes systematically organized into candidate themes through iterative comparison and refinement, supported by thematic maps to visualize relationships between codes. Phase 4 involved rigorous theme review and refinement to ensure internal homogeneity and external heterogeneity. Phase 5 included final theme definition and nomenclature development, while Phase 6 comprised comprehensive report production. Member checking procedures were conducted with representative participant subsets to enhance finding credibility and interpretive authenticity.

Researcher positionality and reflexivity: the research team acknowledges that three members were directly involved in the CREATE model’s implementation, which may have influenced data interpretation. To mitigate this potential bias, coding was independently conducted by two researchers, one of whom had no involvement in the intervention. Regular reflexive discussions were held throughout the analytical process, and an audit trail was maintained documenting all analytical decisions. These measures were implemented to enhance the trustworthiness of the qualitative findings and to ensure that the analysis remained critically engaged rather than confirmatory.

#### Mixed-methods integration

Comprehensive data integration occurred during interpretation phases using joint display matrices to identify convergent, divergent, and complementary findings between quantitative and qualitative data sources. Meta-inferences were systematically developed through careful synthesis of insights from both analytical strands, providing comprehensive understanding of the observed changes and their underlying mechanistic pathways.

## Results

### Participant characteristics and study flow

A total of 86 registered nurses successfully participated in the CREATE model intervention between October 2023 and October 2024, representing a diverse cross-section of clinical specialties, experience levels, and demographic characteristics. Participant retention achieved 100% completion rates, with all enrolled nurses successfully completing both pre- and post-intervention assessment protocols, though this high retention rate should be interpreted in the context of the voluntary and self-selected nature of the sample. Demographic analysis revealed that the majority of participants were female (89.5%, *n* = 77), with ages ranging from 24 to 45 years (mean 31.2 ± 6.8 years). Clinical experience demonstrated substantial variation from 1 to 20 years, with a median of 5.5 years and mean of 7.3 ± 4.9 years. Participants represented 18 different clinical departments throughout the institution, with largest representation from intensive care units (20.9%, *n* = 18), medical-surgical units (18.6%, *n* = 16), and emergency departments (15.1%, *n* = 13). Educational backgrounds included 58.1% (*n* = 50) with bachelor’s degrees, 38.4% (*n* = 33) with associate degrees, and 3.5% (*n* = 3) with master’s degrees. Notably, prior research experience was markedly limited, with only 12.8% (*n* = 11) having previously participated in any research activities and merely 7.0% (*n* = 6) having achieved peer-reviewed publication success (Table 1).

**TABLE 1:** Participant demographic and clinical characteristics (*N* = 86).

Characteristic	*n* (%)	Mean ± SD	Range
Age (years)	–	31.2 ± 6.8	24–45
Gender			
Female	77 (89.5)	–	–
Male	9 (10.5)	–	–
Education level			
Associate degree	33 (38.4)	–	–
Bachelor’s degree	50 (58.1)	–	–
Master’s degree	3 (3.5)	–	–
Clinical experience (years)	–	7.3 ± 4.9	1–20
Clinical department			
Intensive care units	18 (20.9)	–	–
Medical-surgical units	16 (18.6)	–	–
Emergency department	13 (15.1)	–	–
Operating room	10 (11.6)	–	–
Pediatrics	8 (9.3)	–	–
Obstetrics/gynecology	7 (8.1)	–	–
Other specialties	14 (16.3)	–	–
Previous research experience			
Any prior research participation	11 (12.8)	–	–
Previous publications	6 (7.0)	–	–
Grant application experience	3 (3.5)	–	–

### Primary outcome: research competence enhancement

**FIGURE 1:**
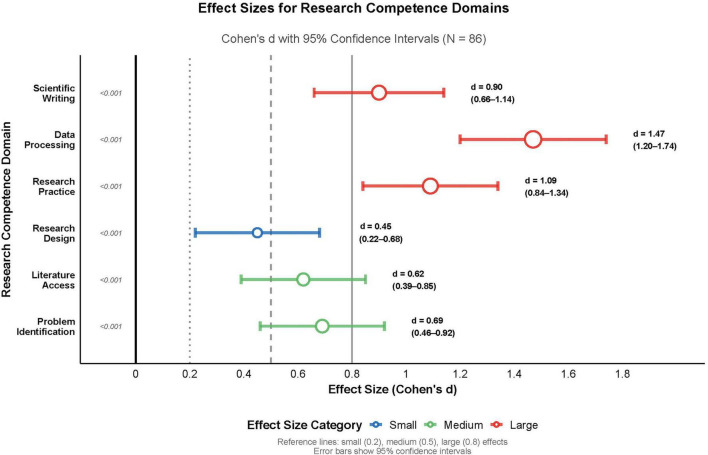
Effect sizes for research competence domains following CREATE model intervention. Forest plot showing Cohen’s d effect sizes with 95% confidence intervals for six research competence domains (*N* = 86). Points are color-coded by effect size: blue (small), green (medium), red (large). Vertical reference lines indicate effect thresholds (0.2, 0.5, 0.8). Data processing (*d* = 1.47) and research practice (*d* = 1.09) showed the largest improvements; research design showed the smallest improvement (*d* = 0.45). All *p* < 0.001.

The primary outcome analysis revealed statistically significant and clinically meaningful improvements in self-reported research competence across all measured domains (Figure 1). Pre-intervention NRCSAS total scores averaged 68.72 ± 18.38, increasing substantially to 84.97 ± 12.95 post-intervention, representing a statistically significant improvement (t(85) = −18.45, *p* < 0.001, Cohen’s *d* = 1.02, 95% CI: 0.78–1.26). This 23.6% increase in total competence scores represents a substantial observed change, though the absence of a control group means that these improvements cannot be definitively attributed to the intervention alone.

Following Bonferroni correction for multiple comparisons across seven tests (six subscales and total score), the adjusted significance threshold was established at *p* < 0.007 (0.05/7). All domain-specific and total score comparisons remained statistically significant at this corrected threshold, as all *p*-values were <0.001. Domain-specific analyses demonstrated notable consistency in improvement patterns across all measured competency areas, as illustrated in the forest plot (Figure 1). Problem identification abilities showed significant enhancement from 8.26 ± 1.67 to 9.37 ± 1.56 (t(85) = −10.75, *p* < 0.001, Cohen’s *d* = 0.69, 95% CI: 0.46–0.92), reflecting participants’ substantially enhanced confidence in recognizing clinically relevant research questions and formulating investigable hypotheses. Literature access and critical appraisal competence increased significantly from 14.90 ± 2.94 to 16.55 ± 2.36 (t(85) = −8.16, *p* < 0.001, Cohen’s *d* = 0.62, 95% CI: 0.39–0.85), indicating markedly improved capabilities in evidence retrieval, database navigation, and critical evaluation methodologies.

Research design competence demonstrated notable improvement from 11.26 ± 4.32 to 13.02 ± 3.52 (t(85) = −8.65, *p* < 0.001, Cohen’s *d* = 0.45, 95% CI: 0.22–0.68), though with a more modest effect size, suggesting this complex methodological domain requires sustained long-term development and continued reinforcement. The most substantial competency gains occurred in research practice implementation, increasing dramatically from 13.21 ± 4.63 to 17.66 ± 3.42 (t(85) = −14.40, *p* < 0.001, Cohen’s *d* = 1.09, 95% CI: 0.84–1.34), and data processing capabilities, which improved remarkably from 10.21 ± 3.66 to 14.74 ± 2.34 (t(85) = −16.63, *p* < 0.001, Cohen’s *d* = 1.47, 95% CI: 1.20–1.74). Scientific writing competence also demonstrated significant improvement from 10.90 ± 3.30 to 13.62 ± 2.70 (t(85) = −10.52, *p* < 0.001, Cohen’s *d* = 0.90, 95% CI: 0.66–1.14) (Table 2).

**TABLE 2:** Pre-post intervention comparison of nursing research competence (*N* = 86).

NRCSAS domain	Pre-intervention mean ± SD	Post-intervention mean ± SD	Mean difference (95% CI)	*T*-value	*P*-value	Cohen’s d (95% CI)
Problem identification	8.26 ± 1.67	9.37 ± 1.56	1.11 (0.90–1.32)	−10.75	<0.001	0.69 (0.46–0.92)
Literature access and appraisal	14.90 ± 2.94	16.55 ± 2.36	1.65 (1.25–2.05)	−8.16	<0.001	0.62 (0.39–0.85)
Research design	11.26 ± 4.32	13.02 ± 3.52	1.76 (1.35–2.17)	−8.65	<0.001	0.45 (0.22–0.68)
Research practice	13.21 ± 4.63	17.66 ± 3.42	4.45 (3.84–5.06)	−14.40	<0.001	1.09 (0.84–1.34)
Data processing	10.21 ± 3.66	14.74 ± 2.34	4.53 (3.99–5.07)	−16.63	<0.001	1.47 (1.20–1.74)
Scientific writing	10.90 ± 3.30	13.62 ± 2.70	2.72 (2.21–3.23)	−10.52	<0.001	0.90 (0.66–1.14)
Total score	68.72 ± 18.38	84.97 ± 12.95	16.25 (14.51–17.99)	−18.45	<0.001	1.02 (0.78–1.26)

All comparisons used paired *t*-tests. Effect sizes: small (*d* = 0.2), medium (*d* = 0.5), large (*d* = 0.8). All *p*-values remained statistically significant following Bonferroni correction for multiple comparisons across seven tests (six subscales and total score). The adjusted significance threshold was *p* < 0.007 (0.05/7); all reported *p*-values were <0.001, well below this corrected threshold.

**FIGURE 2:**
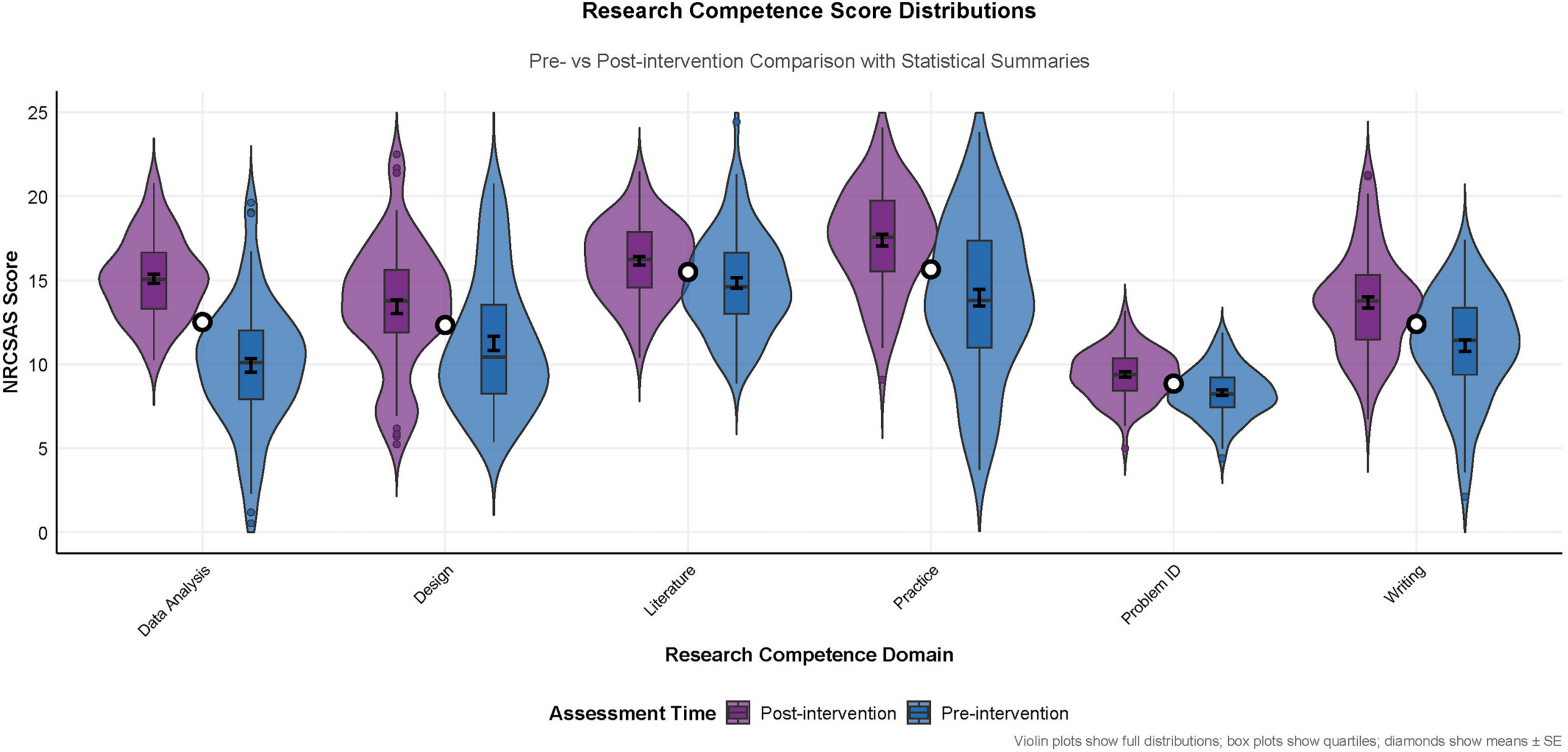
Distribution of research competence scores before and after CREATE intervention. Violin plots with box plot overlays comparing pre-intervention (blue) and post-intervention (purple) score distributions across six competence domains (*N* = 86). White diamonds show means ±SE. The plots demonstrate significant score increases and reduced variability post-intervention across all domains, with particularly notable improvements in data processing and research practice.

The distribution of individual participant scores before and after the intervention further illustrates the comprehensive nature of these improvements (Figure 2). The violin plots demonstrate not only shifts in mean scores but also reduced variability post-intervention, suggesting that improvements were observed across the entire competence spectrum.

Comprehensive subgroup analyses revealed that improvement patterns remained consistent across diverse demographic characteristics, with no statistically significant differences detected based on participant age, accumulated clinical experience, educational background, or departmental affiliation. However, participants with no prior research experience demonstrated substantially larger effect sizes compared to those with previous research exposure (*d* = 1.15 vs. *d* = 0.78, *p* = 0.032), suggesting the CREATE intervention may be particularly beneficial for complete research novices.

### Secondary outcomes: research productivity and scholarly achievement

**FIGURE 3:**
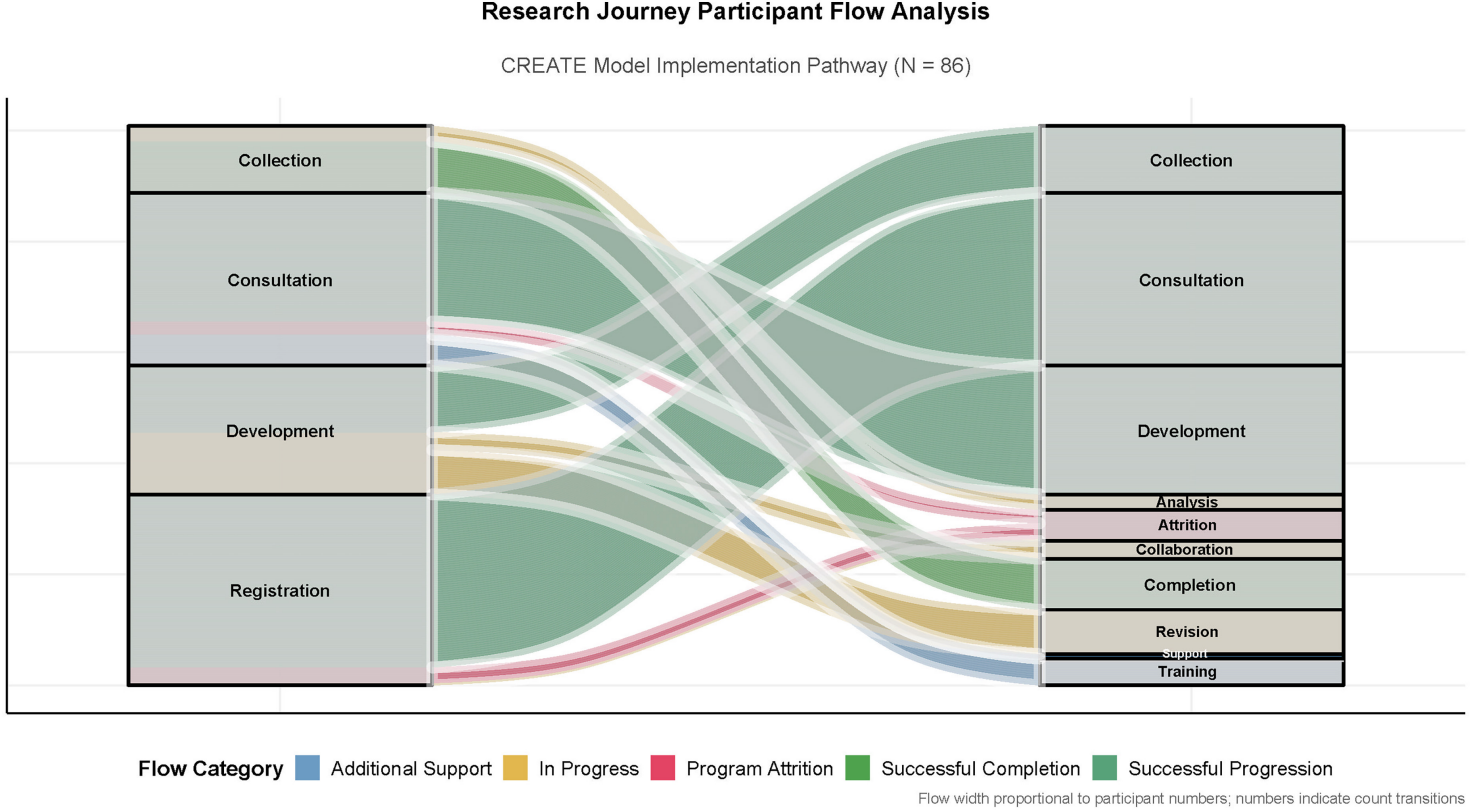
Participant flow through CREATE model research journey. Sankey flow diagram showing participant pathways from registration to completion (*N* = 86). Flow width represents participant numbers; colors indicate outcomes: green (successful progression/completion), blue (additional support), yellow (ongoing), red (attrition). 78 participants reached first consultation, 58 developed proposals, 30 began data collection, and 23 completed projects.

Participation in the CREATE model was associated with tangible improvements in research productivity extending well beyond self-assessed competence measures. Detailed analysis of participant pathways through the intervention reveals the comprehensive journey from initial engagement to research completion (Figure 3). During the comprehensive 12-months study period, participating nurses achieved notable scholarly outputs including one publication in a core-indexed peer-reviewed journal, three oral presentations delivered at national nursing conferences, three utility model patents addressing innovative clinical practice solutions, and four successful competitive applications for institutional youth research fund award totaling ¥180,000 in direct funding support. Additionally, participants successfully initiated 12 comprehensive quality improvement projects within their respective clinical units, with six projects ultimately resulting in measurable process or outcome improvements that were formally integrated into standard practice protocols (Table 3).

**TABLE 3:** Research productivity outcomes during study period (*N* = 86).

Outcome measure	Number	Percentage of participants	Progression rate (denominator)
Scholarly publications			
Core-indexed journal articles	1	1.2%	–
Conference abstracts submitted	8	9.3%	–
Conference presentations delivered	3	3.5%	–
Intellectual property			
Utility model patents filed	3	3.5%	–
Innovation proposals submitted	12	14.0%	–
Grant applications			
Institutional youth fund award	4	4.7%	–
External funding applications	2	2.3%	–
Quality improvement projects			
Projects initiated	12	14.0%	–
Projects completed	6	7.0%	50.0% of 12 initiated
Projects with measurable outcomes	6	7.0%	100% of 6 completed
Research proposals developed	58	67.4%	67.4% of 86 enrolled
Studies advancing to data collection	30	34.9%	51.7% of 58 proposals
Studies completed	23	26.7%	76.7% of 30 in data collection

These productivity outcomes, while modest in absolute terms, should be interpreted as early-stage indicators of research engagement among a cohort in which the vast majority (87.2%) lacked any prior research experience, reflecting the reality that sustainable research productivity requires extended developmental timelines to fully materialize (19).

**FIGURE 4:**
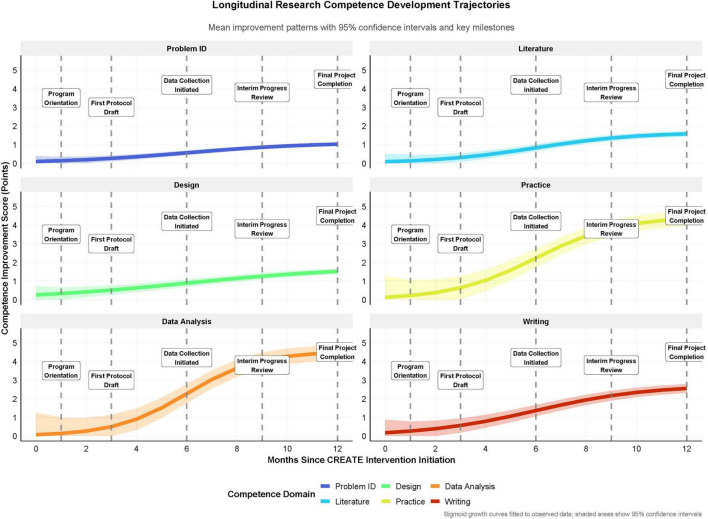
Longitudinal trajectories of research competence development. Sigmoid growth curves showing competence development over 12 months (*N* = 86). Solid lines show mean trajectories; shaded areas show 95% CI. Vertical dashed lines mark intervention milestones. Data processing and research practice domains showed steepest improvement trajectories, while research design demonstrated gradual but sustained development.

Detailed research engagement pattern analysis revealed that 67.4% of enrolled participants (*n* = 58 of 86) successfully developed formal, comprehensive research proposals during the intervention period; of these proposal authors, 51.7% (*n* = 30 of 58) progressed to active data collection. Among the participants reaching the data collection stage, 76.7% (*n* = 23 of 30) successfully completed their studies within the evaluation timeframe. These figures correspond to the progression-rate denominators reported in the right-hand column of Table 3, and are distinct from the participation rates (calculated against the full enrolled cohort of *n* = 86) presented in the same table. The median duration from initial consultation to project completion was 8.2 months (range: 4.5–12.0 months), demonstrating sustained research momentum throughout the intervention period. The longitudinal development of research competencies throughout the intervention period demonstrates distinct growth trajectories for different domains (Figure 4). Data processing and research practice implementation showed the steepest improvement curves, while research design competence exhibited more gradual but sustained development over time.

### Operational performance and participant satisfaction

Comprehensive operational metrics demonstrated robust clinic utilization patterns and consistently high participant engagement levels throughout the study period. A total of 342 individual consultation sessions were successfully conducted, averaging 4.0 sessions per participant (range 2–8 sessions). The specialist provider attendance rate achieved 100% reliability, with zero canceled appointments attributable to provider unavailability throughout the entire study period. Appointment scheduling demonstrated progressive utilization increases, with capacity utilization rates growing systematically from 68% during the initial implementation quarter to 94% by study completion.

Comprehensive satisfaction assessment revealed exceptionally high participant ratings across all measured service dimensions. Overall satisfaction with clinic services averaged 4.6 ± 0.5 on the 5-point evaluation scale, with 94.2% (*n* = 81) of participants rating their comprehensive experience as “satisfied” or “very satisfied.” Mentor effectiveness received the highest satisfaction ratings (4.7 ± 0.4), followed closely by resource adequacy (4.5 ± 0.6) and appointment accessibility (4.4 ± 0.7). When queried regarding likelihood to recommend the clinic services to professional colleagues, 96.5% (*n* = 83) responded “definitely” or “probably yes,” indicating exceptional participant advocacy and program endorsement (Table 4).

**TABLE 4:** Clinic satisfaction and operational metrics.

Metric	Result
Satisfaction ratings (1–5 scale)	Mean ± SD
Overall clinic satisfaction	4.6 ± 0.5
Mentor effectiveness	4.7 ± 0.4
Resource adequacy	4.5 ± 0.6
Appointment accessibility	4.4 ± 0.7
Operational performance	
Total consultation sessions conducted	342
Average sessions per participant	4.0 (range: 2–8)
Specialist attendance rate	100%
Participant retention rate	100%
Recommendation likelihood	*n* (%)
Definitely would recommend	65 (75.6)
Probably would recommend	18 (20.9)
Uncertain	3 (3.5)
Probably/definitely would not recommend	0 (0.0)

### Qualitative findings: understanding transformational mechanisms

Comprehensive thematic analysis of the 20 in-depth participant interviews yielded five major transformational themes that illuminate the underlying mechanisms potentially responsible for quantitative improvements while providing rich contextual understanding of participants’ lived experiences throughout the CREATE model implementation.

Theme 1: From Professional Apprehension to Scholarly Empowerment–A Fundamental Identity Transformation

Participants universally described initiating their clinic engagement with profound apprehension and substantial self-doubt, consistently viewing research as an intimidating intellectual domain exclusively reserved for academics and professional researchers. This initial trepidation was characteristically manifested through feelings of professional inadequacy and impostor syndrome, with numerous participants explicitly expressing beliefs that research represented activities “not intended for people like me” or “beyond my intellectual capabilities.” However, the systematically structured, progressively challenging nature of the CREATE model systematically dismantled these psychological barriers, fostering notable transformations in professional identity conceptualization and research self-efficacy development. This transformation resonates with Bandura’s self-efficacy theory, which posits that mastery experiences, vicarious learning, and verbal persuasion constitute primary sources of efficacy beliefs–all of which were structurally embedded within the CREATE model’s design.

The empowerment transformation evolved through distinct, identifiable developmental stages, beginning with comprehensive demystification of research processes and methodologies, progressing through systematic skill acquisition and confidence building experiences, and ultimately culminating in authentic ownership of scholarly researcher identity. Participants described experiencing profound paradigmatic shifts in their fundamental approaches to clinical problem-solving, evolving from passive acceptance of established practices to proactive inquiry-based engagement. As one intensive care unit nurse eloquently articulated: *“Prior to my clinic engagement, research represented this insurmountable intellectual mountain I believed I could never successfully navigate. Following my CREATE experience, whenever I encounter clinical problems on my unit, my immediate instinct has become asking ‘what does the current evidence demonstrate?’ I have fundamentally transformed into someone who systematically questions, rigorously investigates, and refuses to accept practices simply because ‘that’s how we’ve always approached things.”’*

Theme 2: Mentorship as the Catalytic Mechanism for Professional Growth and Development

The structured mentoring relationship emerged as the single most influential component of the comprehensive CREATE model, significantly transcending traditional teacher-student dynamics to encompass professional advocacy, emotional support, and comprehensive career development guidance. Participants consistently emphasized that effective mentorship extended substantially beyond technical guidance provision to include sustained emotional support, systematic career counseling, and confidence building assistance during challenging phases of their individual research journeys. This finding is consistent with Vygotsky’s concept of the zone of proximal development, whereby learners achieve higher levels of competence through guided interaction with more knowledgeable others than they could independently attain.

The mentorship model’s exceptional effectiveness stemmed directly from its highly individualized approach, with dedicated mentors systematically adapting their guidance methodologies to accommodate participants’ unique learning preferences, accumulated experience levels, and specific professional development goals. Mentors simultaneously fulfilled multiple essential roles: technical advisors providing sophisticated methodological expertise, professional cheerleaders offering sustained encouragement during inevitable setbacks, and institutional advocates facilitating navigation of complex organizational barriers. The sustained longitudinal nature of these professional relationships proved absolutely crucial, as participants consistently valued having reliable, consistent support throughout their complete research processes rather than episodic consultation interactions.

Theme 3: Legitimizing Scholarly Inquiry Through Institutional Recognition and Resource Commitment

The formal organizational establishment of the Nursing Research Clinic transmitted powerful symbolic messaging throughout the entire institutional culture regarding the genuine value placed on nursing scholarly inquiry and intellectual contribution. Participants interpreted the clinic’s existence as tangible, concrete evidence that their intellectual contributions were authentically recognized, valued, and institutionally supported, contrasting dramatically with previous experiences of attempting to conduct research activities as entirely extracurricular, personally funded endeavors. This legitimization process aligns with Senge’s organizational learning theory, which emphasizes the necessity of institutional structures that enable and validate learning at all organizational levels.

This legitimization process operated systematically at multiple organizational levels, beginning with dedicated resource allocation, protected time provision, and expert personnel assignment specifically supporting nursing research activities. The clinic’s formal operational structure, including its sophisticated electronic platform for appointment scheduling and dedicated physical space allocation, symbolized meaningful institutional commitment to nursing scholarship advancement. Participants described experiencing profound liberation from previous needs to “apologize” for pursuing research interests or conducting scholarly activities entirely during personal time away from professional responsibilities.

Theme 4: Breaking Down Institutional Silos Through Collaborative Partnership Development

**FIGURE 5:**
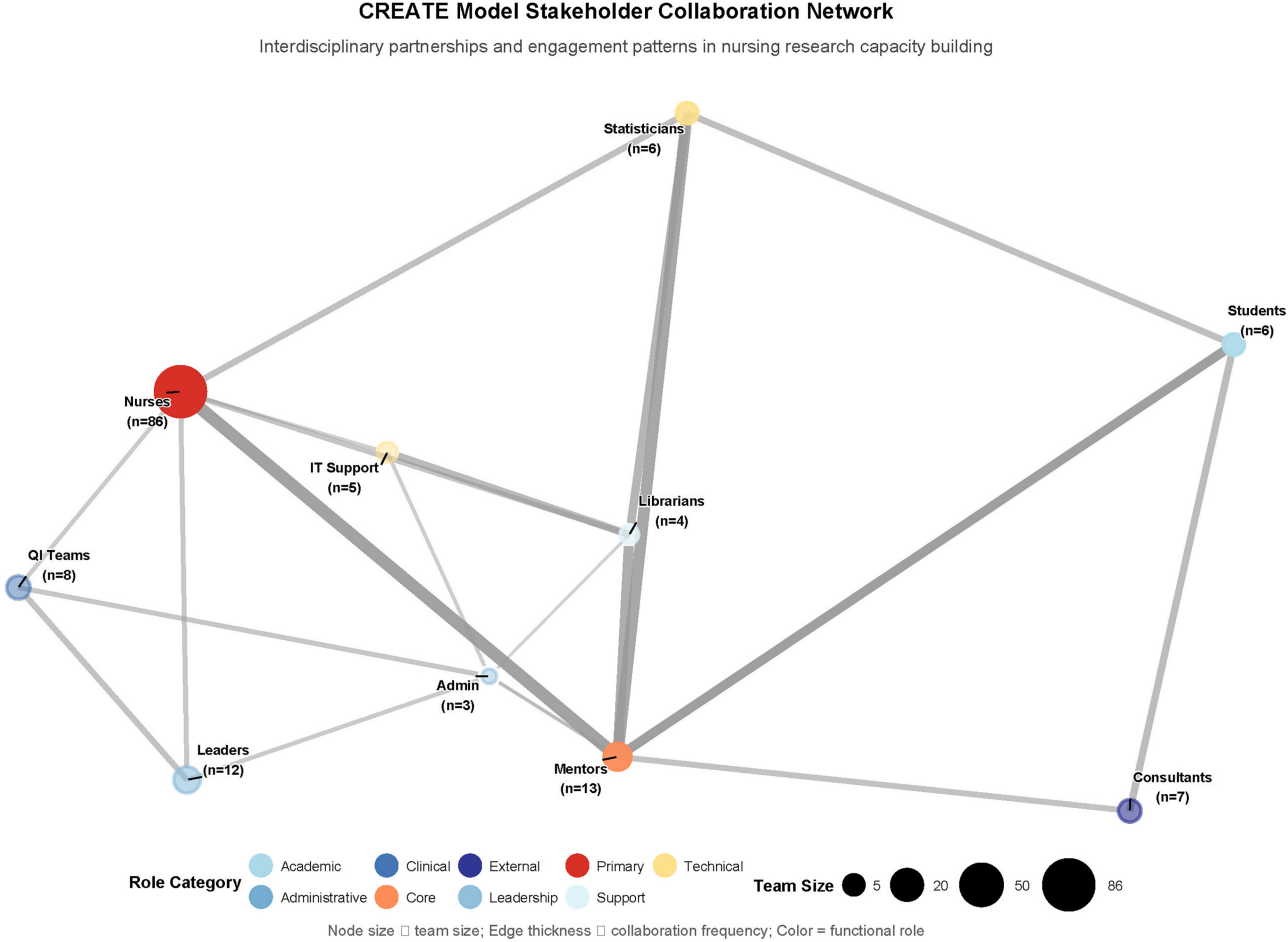
Stakeholder collaboration network in CREATE model implementation. Network diagram of interdisciplinary collaboration patterns. Node size represents team size (*n* = 3–86); colors indicate functional roles; edge thickness shows collaboration strength. The hub-and-spoke pattern centers on clinical nurses and research mentors, with extensive connections to statistical consultants, information specialists, and other support services.

The CREATE model’s emphasis on interdisciplinary collaboration and comprehensive academic-practice partnerships proved transformational in dismantling traditional hierarchical boundaries and departmental operational silos. The network analysis of stakeholder collaboration patterns illustrates the hub-and-spoke structure centered on clinical nurses and research mentors, with extensive connections to statistical consultants, information specialists, and other support services (Figure 5). Participants described how sustained collaboration with specialists, researchers, and colleagues from diverse departments fostered unprecedented mutual respect and collective ownership of patient care improvement through evidence-based innovation implementation.

These collaborative professional relationships evolved substantially beyond project-specific interactions to encompass ongoing knowledge exchange networks and sustained professional networking opportunities. Participants consistently valued opportunities to contribute their specialized clinical insights to research question development while simultaneously learning from academic perspectives and rigorous methodological expertise. This bidirectional knowledge flow systematically enhanced both the clinical relevance of research questions and the methodological rigor of study design implementations.

Theme 5: Navigating Persistent Challenges in Clinical Research Implementation

Despite the CREATE model’s numerous documented successes, participants provided remarkably candid descriptions of ongoing challenges that persisted throughout their complete research journeys. The most frequently cited obstacle involved the complexity gap between expert guidance received during structured consultations and participants’ actual capabilities to independently implement received recommendations. This “implementation translation barrier” frequently resulted from insufficient foundational knowledge in advanced research methodology and sophisticated statistical analysis techniques.

The characteristically high-intensity nature of clinical nursing practice created ongoing tension with the sustained intellectual attention required for meaningful research activities. Participants described cyclical patterns of enthusiasm and frustration, as demanding clinical responsibilities frequently interrupted research momentum and substantially delayed project completion timelines. Many participants expressed legitimate concerns regarding their capacity to maintain research engagement following completion of the structured clinic intervention, recognizing continued need for external support systems and professional accountability mechanisms. Additionally, participants identified substantial challenges in translating completed research findings into actual practice changes within their clinical units, highlighting critical need for additional implementation support extending beyond research completion phases.

## Discussion

This comprehensive mixed-methods evaluation provides evidence suggesting that the CREATE model, systematically operationalized through a dedicated Nursing Research Clinic infrastructure, is associated with meaningful improvements in clinical nurses’ research competencies while fostering sustainable cultures of scholarly inquiry within complex healthcare environments. The quantitative findings demonstrate statistically significant and clinically meaningful improvements across all measured domains of perceived research competence, with large effect sizes suggesting substantial observed change, though the single-group design precludes definitive causal attribution (20). These objective competency improvements are powerfully illuminated and contextualized by the comprehensive qualitative findings, which reveal potential mechanisms through which the intervention may have created protected intellectual space for systematic clinical inquiry while simultaneously addressing both individual knowledge gaps and organizational barriers to sustained research engagement.

The magnitude of research competence score improvement (Cohen’s *d* = 1.02) significantly exceeds effect sizes reported in previous educational interventions targeting nursing research capacity development, suggesting that the CREATE model’s comprehensive structural approach may demonstrate larger observed improvements compared to traditional didactic educational methodologies (21). However, direct comparison is limited by differences in study design, outcome measures, and participant characteristics across studies. The consistency of improvement across all measured competence domains indicates the model’s broad-based reach, though particularly substantial gains in data processing and research practice implementation suggest these practical, applied skills may be especially responsive to intensive mentorship and structured support mechanisms. The significant finding that complete research novices demonstrated larger improvement effects supports the model’s potential accessibility and value for nursing practitioners without prior research exposure.

Examining the quantitative and qualitative findings in an integrated manner reveals several important convergences. The large effect size observed in data processing competence (*d* = 1.47) is contextualized by qualitative accounts of participants’ initial profound fear of statistical analysis and their subsequent confidence growth through structured, individualized mentorship. Similarly, the more modest effect size in research design (*d* = 0.45) aligns with qualitative descriptions of the persistent “translation gap” between expert guidance and independent capability, suggesting that methodological competence requires more sustained developmental support than other domains. The qualitative theme of institutional legitimization provides explanatory context for the high retention rate and sustained engagement observed quantitatively, while the theme of persistent challenges offers important counterbalancing perspective to the uniformly positive statistical findings.

The emergent qualitative theme of “From Apprehension to Empowerment” provides crucial potential mechanistic insight into the underlying processes responsible for that may contribute to these quantitative improvements. The systematic professional identity transformation from traditional clinical practitioner to clinical scholar represents a fundamental paradigmatic shift extending well beyond mere skill acquisition to encompass profound changes in self-perception, professional confidence, and career trajectory conceptualization. This identity transformation process appears absolutely critical for sustaining research engagement beyond formal intervention periods and suggests that effective research capacity building initiatives must systematically address psychological and cultural barriers alongside technical skill development requirements.

The central importance of structured mentorship relationships, as comprehensively revealed through qualitative analysis, aligns closely with established literature demonstrating mentoring relationships as essential mechanisms for professional development and career advancement across healthcare disciplines (19). However, the CREATE model’s primary innovation lies in systematically formalizing and institutionalizing mentorship within permanent organizational structures, thereby ensuring consistent availability, standardized quality, and sustainable delivery. The mentor-mentee relationships described by participants significantly transcended traditional academic advisory roles to encompass comprehensive advocacy, sustained emotional support, and systematic career guidance–a holistic developmental approach that appears crucial for success within demanding clinical environments. This finding corroborates the synthesis by Morrison et al. (6) which identified mentorship, organizational culture, and access to resources as key facilitators of research activity among clinical nurses (21).

The compelling theme of “Legitimizing Inquiry Through Institutional Recognition” reveals a crucial organizational dimension of research capacity building that has received insufficient attention in previous intervention designs and implementations. The formal establishment of a dedicated clinic created visible, tangible institutional commitment to nursing scholarship advancement, systematically transforming research from an individual pursuit to an organizationally supported professional responsibility and expectation. This legitimization appears to address broader cultural barriers that frequently undermine individual-level interventions, suggesting that comprehensive structural changes may represent necessary prerequisites for sustainable research capacity development. These observations are consistent with recent evidence suggesting that structured institutional support, including dedicated mentorship and legitimized research time, constitutes a fundamental enabler of nursing research engagement (7, 22, 23).

The CREATE model is best characterized as a practice-oriented operational framework rather than a theoretical contribution in its own right. It draws upon and operationalizes principles from organizational learning theory (14), self-efficacy theory (24), and academic-practice partnership models, integrating these theoretical foundations into a structured, replicable approach to research capacity building within clinical settings. Its primary value lies in translating established theoretical principles into practical institutional mechanisms–including structured consultation pathways, formalized mentorship, and institutional legitimization of research activity–rather than in advancing theory itself.

Despite these documented successes, this investigation also revealed persistent challenges warranting continued attention and innovative solutions. The identified “translation gap” between expert guidance provision and independent implementation capability, prominently featured through qualitative analysis, suggests that foundational research education remains insufficient for many clinical nursing practitioners. This finding strongly supports calls for enhanced integration of research methodology content in nursing curricula and comprehensive continuing education programming (25). The ongoing tension between intensive clinical demands and research activity requirements highlights urgent need for creative organizational solutions including protected research time allocation and flexible scheduling arrangements. Furthermore, the CREATE model, as implemented at our institution, required substantial resource investment, including a multidisciplinary team of thirteen specialist members, a dedicated electronic platform, and protected consultation time. These resource demands may limit direct transferability to smaller institutions or settings with more constrained budgets, and future research should explore adapted, resource-tiered versions of the model. Perhaps most importantly, this investigation identified limited translation of completed research projects into tangible practice changes–a finding extending beyond the CREATE model’s scope to reflect broader challenges inherent in healthcare implementation science, for which additional strategic approaches are critically needed.

### Limitations

Several methodological limitations warrant consideration. The single-group pre-post design limits internal validity and precludes causal inference, as observed improvements may reflect Hawthorne effects, maturation, regression to the mean, or the motivational effects of voluntary participation rather than the intervention itself; this design was adopted because withholding an institutionally endorsed professional development resource was deemed ethically problematic and a randomized controlled design was not yet feasible at this pilot stage. The reliance on self-reported competence measures captures perceived rather than objectively demonstrated ability, and future studies should incorporate objective assessments such as research proposal quality ratings to complement self-report data. In addition, the adapted version of the instrument used in this study was not separately revalidated within the host institution prior to deployment; while the original Research Competency Scale for Nursing Students by Qiu et al. has documented psychometric properties, formal psychometric evaluation of the adapted item set in registered clinical nurses remains a task for future work. The voluntary participation design introduces selection bias, as enrolled nurses may have been more motivated than non-participating colleagues, and the 100% retention rate may partly reflect this self-selection (17). Additionally, the single-center design constrains generalizability, and the 12-months evaluation period is insufficient to determine whether improvements are sustained over time. Future research should systematically incorporate controlled designs, multi-site evaluation, objective competence measures, and extended follow-up periods to comprehensively establish the model’s effectiveness and replicability.

The comprehensive implications for nursing practice, education, and leadership are substantial. For clinical practice, the CREATE model provides a systematically evaluated, potentially replicable blueprint for empowering frontline nursing practitioners to assume clinical scholar roles while maintaining essential patient care responsibilities. For nursing education, these findings emphasize urgent need for enhanced research literacy integration in foundational nursing curricula and innovative academic-practice partnership development. For nursing leadership, this investigation presents encouraging preliminary evidence supporting strategic investment in comprehensive structural supports for nursing research as a potentially effective organizational strategy for advancing institutional excellence and improving patient care outcomes.

## Conclusion

This evaluation suggests that bridging the persistent theory-to-practice gap requires substantially more than individual education and training–it demands systematic, comprehensive organizational commitment to creating supportive environments for sustained clinical inquiry. The CREATE model offers a promising framework for healthcare institutions seeking to advance nursing excellence and enhance organizational research capacity. The convergent quantitative and qualitative evidence presented herein demonstrates meaningful improvements in research competence, early-stage scholarly productivity, and professional identity transformation, supported by structured mentorship, institutional legitimization, and interdisciplinary collaboration. While the model’s effectiveness must be confirmed through rigorous controlled evaluation in diverse settings, the present findings provide a robust foundation for future developments in nursing research capacity building and organizational culture transformation.

## Data Availability

The original contributions presented in this study are included in this article/supplementary material, further inquiries can be directed to the corresponding author.
